# Supplementary material for: Does livestock ownership predict animal-source food consumption frequency among children aged 6–24 months and their mothers in the rural Dale district, southern Ethiopia?
Source: PeerJ. 2023 Dec 14;11:e16518. doi: 10.7717/peerj.16518 (PMC10725678; doi:10.7717/peerj.16518)
Supplement: Supplemental Information 2 [file peerj-11-16518-s002.docx]

**Supplemental Table S1: Distribution of animal-source foods consumption among children aged 6-24 months by household livestock ownership the Dale district, southern Ethiopia (N=851).**

| Livestock ownership | | Animal source foods consumed during the month prior to our survey | | | | | | | | |
| --- | --- | --- | --- | --- | --- | --- | --- | --- | --- | --- |
|  |  | Dairy | | | Eggs | | | Meat | | |
|  |  | Yes | No | Total | Yes | No | Total | Yes | No | Total |
| Cow | Yes | 577 (91.4%) | 54 (8.6%) | 631 | 531 (84.2%) | 100 (15.8%) | 631 | 155 (24.6% ) | 476 (75.4%) | 631 |
|  | No | 204 (92.7%) | 16 (7.3%) | 220 | 175 (79.5%) | 45 (20.5%) | 220 | 68 (30.9%) | 152 (69.1%) | 220 |
| Goat/sheep | Yes | 199 (93.4%) | 14 (6.6%) | 213 | 191 (89.7%) | 22 (10.3%) | 213 | 61 (28.6%) | 152 (71.4%) | 213 |
|  | No | 582 (91.2%) | 56 (8.8%) | 638 | 515 (80.7%) | 123 (19.3%) | 638 | 162 (25.4%) | 476 (74.6%) | 638 |
| Hen | Yes | 473 (93.3%) | 34 (6.7%) | 507 | 461 (90.9%) | 46 (9.1%) | 507 | 139 (27.4%) | 368 (72.6%) | 507 |
|  | No | 308 (89.5%) | 36 (10.5%) | 344 | 245 (71.2%) | 99 (28.8%) | 344 | 84 (24.4%) | 260 (75.6%) | 344 |
