# Supplementary material for: Does livestock ownership predict animal-source food consumption frequency among children aged 6–24 months and their mothers in the rural Dale district, southern Ethiopia?
Source: PeerJ. 2023 Dec 14;11:e16518. doi: 10.7717/peerj.16518 (PMC10725678; doi:10.7717/peerj.16518)
Supplement: Supplemental Information 3 [file peerj-11-16518-s003.docx]

**Supplemental Table S2: Distribution of animal-source foods consumption among mothers by household livestock ownership the Dale district, southern Ethiopia (N=851).**

| Livestock ownership | | Animal source foods consumed during the month prior to our survey | | | | | | | | |
| --- | --- | --- | --- | --- | --- | --- | --- | --- | --- | --- |
|  |  | Dairy | | | Eggs | | | Meat | | |
|  |  | Yes | No | Total | Yes | No | Total | Yes | No | Total |
| Cow | Yes | 615 (97.5%) | 16 (2.5%) | 631 | 340 (53.9%) | 291 (46.1%) | 631 | 239 (37.9%) | 392 (62.1%) | 631 |
|  | No | 202 (91.8%) | 18 (8.2%) | 220 | 81 (36.8%) | 139 (63.2%) | 220 | 50 (22.7%) | 170 (77.3%) | 220 |
| Goat/sheep | Yes | 207 (97.2%) | 6 (2.8%) | 213 | 146 (68.5%) | 67 (31.5%) | 213 | 108 (50.7%) | 105 (49.3%) | 213 |
|  | No | 610 (95.6%) | 28 (4.4%) | 638 | 275 (43.1%) | 363 (56.9%) | 638 | 181 (28.4%) | 457 (71.6%) | 638 |
| Hen | Yes | 491 (96.8%) | 16 (3.2%) | 507 | 301 (59.4%) | 206 (40.6%) | 507 | 208 (41.0%) | 299 (60.0%) | 507 |
|  | No | 326 (94.8%) | 18 (5.2%) | 344 | 120 (34.9%) | 224 (65.1%) | 344 | 81 (23.5%) | 263 (76.5%) | 344 |
