# Supplementary material for: Does livestock ownership predict animal-source food consumption frequency among children aged 6–24 months and their mothers in the rural Dale district, southern Ethiopia?
Source: PeerJ. 2023 Dec 14;11:e16518. doi: 10.7717/peerj.16518 (PMC10725678; doi:10.7717/peerj.16518)
Supplement: Supplemental Information 4 [file peerj-11-16518-s004.docx]

**Supplemental Table S3: Animal-source food consumption in the past month as compared between male and female children and mothers in Dale district, southern Ethiopia (N=851).**

| **Animal-source foods** |  | **Consumed**  **N (%)** | **Not consumed**  **N (%)** | **Total**  **N (%)** |
| --- | --- | --- | --- | --- |
| Milk and milk products | Children (female) | 394 (91) | 39 (9) | 433 (100) |
|  | Children (male) | 387 (92.6) | 31 (7.4) | 418 (100) |
|  | Children (all) | 781 (91.8) | 70 (8.2) | 851 (100) |
|  | Mothers | 817 (96.0) | 34 (4.0) | 851 (100) |
| Eggs | Children (female) | 359 (82.9) | 74 (17.1) | 433 (100) |
|  | Children (male) | 347 (83) | 71 (17) | 418 (100) |
|  | Children (all) | 706 (83) | 145 (17) | 851 (100) |
|  | Mothers | 421 (49.4) | 430 (50.6) | 851 (100) |
| Meat (flesh, organs, poultry and fish) | Children (female) | 111 (25.6) | 322 (74.4) | 433 (100) |
|  | Children (male) | 112 (26.8) | 306 (72.2) | 418 (100) |
|  | Children (all) | 223 (26.2) | 628 (73.8) | 851 (100) |
|  | Mothers | 197 (23.1) | 654 (76.9) | 851 (100) |
| Any animal-source foods | Children (female) | 402 (92.8) | 31 (7.2) | 433 (100) |
|  | Children (male) | 393 (94) | 25 (6) | 418 (100) |
|  | Children (all) | 795 (93.4) | 56 (6.6) | 851 (100) |
|  | Mothers | 826 (97.1) | 25 (2.9) | 851 (100) |
