# Supplementary material for: Does livestock ownership predict animal-source food consumption frequency among children aged 6–24 months and their mothers in the rural Dale district, southern Ethiopia?
Source: PeerJ. 2023 Dec 14;11:e16518. doi: 10.7717/peerj.16518 (PMC10725678; doi:10.7717/peerj.16518)
Supplement: Supplemental Information 5 [file peerj-11-16518-s005.docx]

**Supplemental Table S4: Animal-source food consumption among child-mother pairs by households within the past one month in Dale district, southern Ethiopia (N=851).**

| **Animal-source foods** | **Consumption among child-mother pairs in the household** | **Frequency (N)** | **Percentage (%)** |
| --- | --- | --- | --- |
| Milk and milk products | Both child and mother consumed | 757 | 88.9 |
|  | Only the child consumed | 24 | 2.8 |
|  | Only the mother consumed | 60 | 7.1 |
|  | Neither of them consumed | 10 | 1.2 |
| Eggs | Both child and mother consumed | 400 | 47.0 |
|  | Only the child consumed | 306 | 35.9 |
|  | Only the mother consumed | 21 | 2.5 |
|  | Neither of them consumed | 124 | 14.6 |
| Meat (flesh, organs, poultry and fish) | Both child and mother consumed | 111 | 13.0 |
|  | Only the child consumed | 112 | 13.2 |
|  | Only the mother consumed | 178 | 20.9 |
|  | Neither of them consumed | 450 | 52.9 |
| Any animal-source foods | Both child and mother consumed | 773 | 90.8 |
|  | Only the child consumed | 22 | 2.6 |
|  | Only the mother consumed | 53 | 6.2 |
|  | Neither of them consumed | 3 | 0.4 |
