# Supplementary material for: Does livestock ownership predict animal-source food consumption frequency among children aged 6–24 months and their mothers in the rural Dale district, southern Ethiopia?
Source: PeerJ. 2023 Dec 14;11:e16518. doi: 10.7717/peerj.16518 (PMC10725678; doi:10.7717/peerj.16518)
Supplement: Supplemental Information 6 [file peerj-11-16518-s006.docx]

**Questionnaire (Sidaamu Afoo version)**

Tini xa’mo dirinsa 24 agani woroo no qaaqquliranna dirinsa 15 - 49 mereero no amuwi itate gara kikkisate kaa’litanno taje xinqate qixeesinoonte. Xa’mubba aantinotta sase kifile amaddinoha ikkana xa’mo xa’matenna taje borreesate injaanno gede mitte mittente kifilera massago uyinoonni

Xa’mubbate badooshshi ______________

**Umikki kifile**

**Maatete xaphphoomu gara la’ano xa’mubba**

1. Minu badooshshe

| 001 | Barra ___/___/____(barra/agana/diro) |
| --- | --- |
| 002 | Ollaa ________________ |
| 003 | Nafara _______________ |
| 004 | Minu badooshshi kiiro ______ |
| 005 | Xa’mote badooshshi kiiro:_______ |
| 006 | Taje xinqannohu badooshshi kirro:__________ |
| 007 | Towaatannohu kiiro:________ |

1. Dagoomunna miinju xa’mubba

| 101 | Konne mine xaphphoomunni meu manni hee’ranno/dirunninna koo/ Teenni bade borreessi/ | Diro | Labaaha | Meyaata |  |
| --- | --- | --- | --- | --- | --- |
|  |  | 5 diri woro |  |  |  |
|  |  | 5-18 diri geeshsha |  |  |  |
|  |  | 18-65 diri geeshsha |  |  |  |
|  |  | 65 diri ale |  |  |  |
|  |  | Xaphphooma |  |  |  |
| 102 | Uyiinoonni doorshi giddonni, minekki nooha kuli (mittu ale doora dandiinanni) | 1. Televizhine _____ 2. Firiije_____ 3. Milli yitanno bilbilla_____ 4. Moteri biskiliite_____ 5. Biskiliite _____ 6. Kameelu_____ 7. Noohu dino_____ | | |  |
| 103 | Uyiinoonni doorshi giddonni, minekki nooha kuli/mittu ale doora dandiinanni | 1. Lukkuwa _____ 2. Me’’e _____ 3. Gerecho _____ 4. Adote saa _____ 5. Booto woyi hando _____ 6. Haricho _____ 7. Farasho _____ | | |  |

2. Xaphphoomu maatete itate gara

| 201 | Maatete duuchchu woyiti sagale maati | 1. Waasa _____ 2. Badala _____ 3. Dinnichcha, baxaaxeessa, bohenna wole rumudaame sagale _____ 4. Wolu hee’riro kuli _______________ | |  |
| --- | --- | --- | --- | --- |
| 202 | Maatete hoollanni sagale no? | 1. Dino _____   2. Ee no _____ | |  |
| 203 | Dawaro ee ikkituro hoollanni sagalenna korkaata kuli | Sagalete dana | Hooloonni korkaata |  |
|  |  |  |  |  |
|  |  |  |  |  |
|  |  |  |  |  |
| 204 | Qaaqqulleho hoollanni sagale no? | 1. Dino _____ 2. Ee no _____ | |  |
| 205 | Dawarokki ee ikkituro, hoollanni sagalenna hoollanni korkaata kuli | Sagalete dana | Hooloonni korkaata |  |
|  |  |  |  |  |
|  |  |  |  |  |
|  |  |  |  |  |
| 206 | Mine amuwaho, godowinni nooriranna qansi’rano amuwira hoollanni sagale no | 1. Dino _____  2. Ee no _____ | |  |
| 207 | Dawaro ee ikkituro hooloonni sagalenna hooloonni korkaata kuli | Sagalete dana | Hooloonni korkaata |  |
|  |  |  |  |  |
|  |  |  |  |  |
|  |  |  |  |  |
|  |  |  |  |  |
| 208 | Maatete wayii buichcho mamaati? | 1. Baanba  2. Fano balenni  3. Diwantino balenni  4. Ha’ranno wayiinni, buichchotenni  5. Wolu hee’riro kuli _____________ | |  |

3. **Sa’u shoolle lamala giddo sagaleteni umo dandaa laino xa’mo**

| 301 | Sai mittu again giddo maatete ikkitanno sagale dino yite qarrante egenootta? | 0. Diegenoomma  1. Ee | Dawaro diegenoomma ikkituro, X. K. 303 sai |
| --- | --- | --- | --- |
| 302 | Sai agani giddo kuni gari mageeshshi yannara iilli? | 1. Sae sae (mitte woyi lame hige) ___  2. Mitte mitte hige (sauyi shoolu geeshsha) ___  3. Duuchcha hige (Tonnu ale) ___ |  |
| 303 | Sai again giddo ati woyi maatekkinni mittu hoongunni kainohunni sagale itate hasidhine ittinikki gattine egentinoonni? | 0. Diegenninoommo  1. Ee | Dawarokki diegeninoommo ikkituro, X. K. 305 sai |
| 304 | Sai again giddo kunirichchi mageeshshi yannara kalaqami? | 1. Sae sae (mitte woyi lame hige) ___  2. Mito mito woyiite (3 - 4 hige) ___  3. Duuchcha hige (tonnu ale) ___ |  |
| 305 | Sai mittu again giddo ati woyi maatekki mereero hoongunni kainohunni gama sagale calla qoltine qoltine ittine egentinoonni? | 0. Diegenninoommo  1. Egenninoommo | Dawarokki diegeninoommo ikkiro X. K. 307 sai |
| 306 | Sai aganira kunirichchi mageeshshi yannara ikki? | 1. Sae sae (mitte woyi lame hige) ___  2. Mito mito woyite (3 – 4 hige) ___  3. Duuchcha hige (tonnu ale) ___ |  |
| 307 | Sai mittu again giddo ati woyi maatekkinni mittu wole sagale afi’ra dandiitinoonnikkihura horonta itate hasidhinannikki sagale ittine egentinoonni? | 0. Diegeninoommo  1. Egeninoommo | Dawarokki diegeninoommo ikkituro X. K. 309 sai |
| 308 | Sai again giddo kunirichchi mageeshshi yannara ikki? | 1. Sae sae (mitte woyi lame hige) ___  2. Mitto mitto woyiite (3 – 4 hige) ___  3. Duuchcha hige (tonnu ale) ___ |  |
| 309 | Sai mittu agani giddo ati woyi maatekkinni mittu ikkitanno sagale nookkihura barrunni ita noonke yitine heddinannihuni ajjino sagale ittine egentinoonni? | 0. Diegeninoommo  1. Egeninoommo | Dawarokki diegeninoommo ikkituro, X. K. 311 sai |
| 310 | Sai again giddo kunirichchi mageeshshi yannara ikki? | 1. Sae sae (mitte woyi lame hige) ___  2. Mito mito woyite (3 – 4 hige) ___  3. Duuchcha hige (tonnu ale) ___ |  |
| 311 | Sai mittu again giddo ati woyi maatekkinni mittu mine ikkitanno sagale hoogatenni barra wo’ma shiima sagale calla ittine egentinoonni? | 1. Diegeninoommo  2. Egeninoommo | Dawarokki diegeninoommo ikkiro, X. K. 313 sai |
| 312 | Sai again giddo kunirichchi mageeshshi yannara ikki? | 1. Sae sae (mitte woyi lame hige) ___  2. Mito mito woyiite (3 – 4 hige) ___  3. Duuchcha hige (tonnu ale) ___ |  |
| 313 | Sai mittu agani giddo hoongunni kainohunni minekki mitturino intanni sagale ba’e egentino? | 1. Diegentino  2. Egentino | Dawarokki diegentino ikkituro, X. K. 315 sai |
| 314 | Sai again giddo kunirichchi mageeshshi yannara ikki? | 1. Sae sae (mitte woyi lame hige) ___  2. Mito mito woyite (3 – 4 hige) ___  3. Duuchcha hige (tonnu ale) ___ |  |
| 315 | Sai mittu again giddo ati woyi maatekkinni mittu ikkitanni sagale hoogatenni hurbaate ittinikki goxxine egentinoonni? | 1. Diegeninoomme  2. Egeninoommo | Dawarokki diegeninoommo ikkituro, X. K. 317 sai |
| 316 | Sai agani giddo kunirichchi mageeshshi yannara ikkI? | 1. Sae sae (mitte woyi lame hige) ___  2. Mito mito woyite (3 – 4 hige) ___  3. Duuchcha hige (Tonnu ale) ___ |  |
| 317 | Sai mittu again giddo ati woyi maatekkinni mittu barra wo’ma sagale horo ittinikki gattine egentinoonni? | 1. Diegenninoommo  2. Egeninoommo | Dawaro diegenninoommo ikkituro, X. K. 319 sai |
| 318 | Sai agani giddo kunirichchi mageeshshi yannara ikki? | 1. Sae sae (mitte woyi lame hige) ___  2. Mito mito woyite (3 – 4 hige) ___  3. Duuchcha hige (tonnu ale) ___ |  |
| 319 | Be’ro barra minekki woyi ollaaho ayyaana woyi baxxino barra ikkinohura albiwi baxxino itate gari no? | 1. Ee 2. Dino |  |

**Layiinkki kifile**

**Xiinxallote eate dooramannoha dirisi 24 agani woroonni ikkino qaaqqo la’ano xa’mubba**

4. Qaaqqu xaphphoomu tajenna itate gara

|  | Qaaqqu koo/tee | Labbaaho________  Meyaate________ |  |
| --- | --- | --- | --- |
|  | Xa’minanni manchi qaaqqu ledo noosi fiixoomi gara | 1. Iltinno ama 2. Ilinno anna 3. Buddeenu ama 4. Buddeenu anna 5. Wolu hee’riro kuli___________ |  |
|  | Diro | ________ aganna |  |
|  | Qaaqqu ilamino barra | ___/___/______(barra/agana/diro) |  |
|  | Qaaqqu ilamino barri kittibaatete/ilamate kaardenni buuxamino | 1. Ee ______ 2. Dibuuxamino ______ |  |
|  | Kuni qaaqqi mama ilamino | 1. Mini giddo ___ 2. Fayyimate uurrinshara (xawisi) ____________ 3. Wolu hee’riro kuli ___________ |  |
|  | Qaaqqu unuuna qananno | 1. Ee qananno ______ 2. Diqananno ______ |  |
|  | Dawarokki diqananno ikkituro, korkaata kuli | 1. Amate faayyima ledo amadaminoha  2. Amate loosi ledo Amadaaminoha  3. Qaaqu faayyimaa ledo Amadaaminoha.  4. Qaaqu Amate unuunin duuwa  5. Amatewin baxiro  6. xa godowin hedhuuro  7. Wolu heriro kuli |  |
|  | Qaaqqu ilamihunni mageeshshi yanna gedensaanni qani | 1. Ilamanni hee’re 2. Ilamihunni mitte sa”ate giddo 3. Ilamihunni mitte sa”ate gedensaanni 4. Ilamihunni lemiina shoole sa”ate gedensaanni 5. Wolu hee’riro kuli __________ |  |
|  | Qaaqqu ilamanni hee’reenna unuunu gobaanni woluri uyinoonnisiri no? | 1. Ee 2. Uyiinoonnisiri dino |  |
|  | Dawarokki ee ikkituro, uyiinoonnisiri maatiro kuli | 1. Bulleete ado ______ 2. Saadate ado______ 3. Waanna sukaare______ 4. Wolu hee’riro kuli __________ |  |
|  | Qaaqqu xuunxo qanino/qane egennino? | 1. Ee______ 2. Qane diegenino______ |  |
|  | Qaaqu unuunu gobbanni wole la’aato adha/ita hanafinno? | 1. Ee ___ 2. Dino ___ |  |
|  | Dawaro e’e ikkituro me’u aganisinni/dirisiniiti qaaqu wole la’aato adha/ita hanafinno? | ________ aganna |  |

1. Qaaqqu itate taalle lainohunni

|  | Kuloonni sagalera ateta itate ledo kulanno yite hedattaha kuli | | | | | | | |
| --- | --- | --- | --- | --- | --- | --- | --- | --- |
|  | **Sagalete dana** | | **Wo’ma wote**  (ajayi ajeenna barrunni mitte hige) | **Roore yanna**  (mitte lamala giddo mitteege kaayise lee hige) | **Sa’ne sa’ne** (mittu aganni giddo mittunni kaayise saseege) | **Taa’i taaiita** (mittu agani giddo mitteege woroonni) | **Horontanni** (sasu aganni giddo horontanni di’itinno) | **Ledote hedo heedhuro** |
| 501 | Adonna adote guma | |  |  |  |  |  |  |
| 502 | Saadate maala (Afale, Mule, WKL) | |  |  |  |  |  |  |
| 503 | Saadate maalanna lukote guma (maalana quuphe) | |  |  |  |  |  |  |
| 504 | Qulxxu’me | |  |  |  |  |  |  |
| 505 | Wole saadate afi’nanni sagallla aleenni xawinsoonikita | |  |  |  |  |  |  |
| **Kulooniri gobbaani itoottari nooro kuli** | | |  |  |  |  |  |  |
| 1 | |  |  |  |  |  |  |  |
| 2 | |  |  |  |  |  |  |  |
| 3 | |  |  |  |  |  |  |  |

**Sayikki kifile**

**Xiinxallote eate doorantinori dirinsa15-49 ikkino meento la’ano xa’mubba**

1. Xaphphomu tajena ilate gara lainohunni

Uyinoonni doorshi giddo ate dawaro albaanni malaate assi woyi uyinoonnihe darga dawaro wonsh

|  |  |  |  |
| --- | --- | --- | --- |
| 601 | Dirikki me”eho | ________ diro |  |
| 602 | Gudoottati jawiidi rosi deerra | 1. Horo rosse egentinkkiti _____ 2. kifile deerra guddinoti _____ 3. Sertifikeetete/dipiloomunni maassantino _____ 4. Digirenna hakuyi ali rosu deerra _____ |  |
| 603 | Damoozunni qaxaramoottoho | Dee’ni _____  Ee _____ |  |
| 604 | Me”e higge godowootta? | __________ |  |
| 605 | Umo godowitta waro dirikki me”eho? | _________ diro |  |
| 606 | Xa buuxamino godowi no? | 1. Ee _____ 2. Dino _____ 3. Diafoomma ________ |  |

1. Amma itate taalle lainohunni

|  | Kuloonni sagalera ateta itate ledo kulanno yite hedattaha kuli | | | | | | | |
| --- | --- | --- | --- | --- | --- | --- | --- | --- |
|  | **Sagalete dana** | | **Wo’ma wote**  (ajayi ajeenna barrunni mitte hige) | **Roore yanna**  (mitte lamala giddo mitteege kaayise lee hige) | **Sa’ne sa’ne** (mittu aganni giddo mittunni kaayise saseege) | **Taa’i taaiita** (mittu agani giddo mitteege woroonni) | **Horontanni** (sasu aganni giddo horontanni di’itinno) | **Ledote hedo heedhuro** |
| 501 | Adonna adote guma | |  |  |  |  |  |  |
| 502 | Saadate maala (Afale, Mule, WKL) | |  |  |  |  |  |  |
| 503 | Saadate maalanna lukote guma (maalana quuphe) | |  |  |  |  |  |  |
| 504 | Qulxxu’me | |  |  |  |  |  |  |
| 505 | Wole saadate afi’nanni sagallla aleenni xawinsoonikita | |  |  |  |  |  |  |
| **Kulooniri gobbaani itoottari nooro kuli** | | |  |  |  |  |  |  |
| 1 | |  |  |  |  |  |  |  |
| 2 | |  |  |  |  |  |  |  |
| 3 | |  |  |  |  |  |  |  |
