# Supplementary material for: Does livestock ownership predict animal-source food consumption frequency among children aged 6–24 months and their mothers in the rural Dale district, southern Ethiopia?
Source: PeerJ. 2023 Dec 14;11:e16518. doi: 10.7717/peerj.16518 (PMC10725678; doi:10.7717/peerj.16518)
Supplement: Supplemental Information 7 [file peerj-11-16518-s007.docx]

**Questionnaire (English version)**

Questionnaire prepared to gather selected socio-demographic, nutrition, and health status information about children aged below 24 months, mothers aged 15- 49 years. The questionnaire contains three sections and directions in each part.

Question identification number _____________

**Section one**

**Questions about general condition of the household**

0. House identification

| 001 | Date / / (day/ month/ year) |
| --- | --- |
| 002 | Kebele |
| 003 | Village/sub-kebele |
| 004 | House identification number |
| 005 | Question identification number |
| 006 | Data collector identification number |
| 007 | Supervisor identification number |

1. Household background

| 101 | How many people live in this house? (identify age and sex) | Year | Male | Female |  |
| --- | --- | --- | --- | --- | --- |
|  |  | < 5 years |  |  |  |
|  |  | 5-18 years |  |  |  |
|  |  | 18-65 years |  |  |  |
|  |  | 65 years and above |  |  |  |
|  |  | Total |  |  |  |
| 102 | Choose from the given properties available in your house (You can choose more than one) | 1.Television ________  2.Refrigator _________  3.Mobile phones _______  4. Motor bike _______  5. Bicycle ________  6. Car ______  7. Nothing available ________ | | |  |
| 103 | Choose from the given properties available in your house (You can choose more than one) | 1. Hens _____ 2. Goat _____ 3. Sheep _____ 4. Cows _____ 5. Bulls/Ox _____ 6. Donkey _____ 7. Horse _____ | | |  |

1. Feeding habit of the family

| 201 | What is the common food used by the family? | 1. Ensete/ ‘Kocho’______ 2. Maize/corn _____ 3. Potato, Sweet potato, ‘Boina’ and other root foods _____ 4. Other/ write ______ | | | |  |
| --- | --- | --- | --- | --- | --- | --- |
| 202 | Is there any food taboo in the family? | 1. No _____ 2. Yes _____ | | | |  |
| 203 | If your answer is yes, describe the type of food and reason for the taboo? | Type of food | What is the reasons | | |  |
|  |  |  |  | | |  |
|  |  |  |  | | |  |
|  |  |  |  | | |  |
| 204 | Is there any food taboo to children? | 1. No _____ 2. Yes _____ | | | |  |
| 205 | If your answer is yes, describe the type of food and reason for the Prohibition? | Type of food | | What is the reason | |  |
|  |  |  | |  | |  |
|  |  |  | |  | |  |
|  |  |  | |  | |  |
| 206 | Is there any food taboo to mothers, pregnant and breast feeding mothers? | 1. No _____ 2. Yes _____ | | | |  |
| 207 | If your answer is yes, describe the type of food and reason for the Prohibition? | Type of food | | | Reason |  |
|  |  |  | | |  |  |
|  |  |  | | |  |  |
|  |  |  | | |  |  |
| 208 | What is common water source of the family? | 1. Pipe water 2. Open pond 3. Closed pond 4. Running river 5. Other/ write   ____________ | | |  |  |

1. Household’s food insecurity access scale (HFIAS) measurement

|  | Did you trouble because there is no enough food to the family in the past one month? | 1. No, I don’t 2. Yes, I do | If your answer is no, go to Q.N. 303 |
| --- | --- | --- | --- |
|  | How long the problem sustained in the past one month? | 1.Occasionally ( one or two times ) ___  2. Sometimes ( three to four times) ___  3. Many times ( above ten times) ___ |  |
|  | Did you or one of your family members missed a meal because of absence of food even if you want to eat in the past one month? | 1. No, I don’t 2. Yes, I do | If your answer is no, go to Q.N. 305 |
|  | How long the problem sustained in the past one month? | 1.Occasionally ( one or two times ) ___  2. Sometimes ( three to four times) ___  3. Many times ( above ten times) ___ |  |
|  | Did you or one of your family members eat the same type of meal repeatedly because of absence of food even if you want to eat in the past one month? | 1. No, I don’t 2. Yes, I do | If your answer is no, go to Q.N. 307 |
|  | How long the problem sustained in the past one month? | 1.Occasionally ( one or two times ) ___  2. Sometimes ( three to four times) ___  3. Many times ( above ten times) ___ |  |
|  | Did you or one of your family members eat a meal you never want to eat because of absence of food in the past one month? | 1. No, I don’t 2. Yes, I do | If your answer is no, go to Q.N. 309 |
|  | How long the problem sustained in the past one month? | 1.Occasionally ( one or two times ) ___  2. Sometimes ( three to four times) ___  3. Many times ( above ten times) ___ |  |
|  | Did you or one of your family members eat small amount of meal even if you want more because of shortage of food in the past one month? | 1. No, I don’t 2. Yes, I do | If your answer is no, go to Q.N. 311 |
| 310 | How long the problem sustained in the past one month? | 1.Occasionally ( one or two times) ___  2. Sometimes ( three to four times) ___  3. Many times ( above ten times) ___ |  |
| 311 | Did you or one of your family members eat small amount of meal within a day because of shortage of food in the past one month? | 1. No, I don’t 2. Yes, I do | If your answer is no, go to Q.N. 313 |
| 312 | How long the problem sustained in the past one month? | 1.Occasionally ( one or two times) ___  2. Sometimes ( three to four times) ___  3. Many times ( above ten times) ___ |  |
| 313 | Did you experience total absence of food in the house because of deprivation in the past one month? | 1. No, I don’t 2. Yes, I do | If your answer is no, go to Q.N.315 |
| 314 | How many times the problem occurs in the past one month? | 1.Occasionally ( one or two times) ___  2. Sometimes ( three to four times) ___  3. Many times ( above ten times) ___ |  |
| 315 | Did you or one of your family members missed diner because of absence of food in the house in the past one month? | 1. No, I don’t 2. Yes, I do | If your answer is no, go to Q.N. 317 |
| 316 | How many times the problem occurs in the past one month? | 1.Occasionally ( one or two times) ___  2. Sometimes ( three to four times) ___  3. Many times ( above ten times) ___ |  |
| 317 | Did you or one of your family members missed a meal the whole day in the past one month? | 1. No, I don’t 2. Yes, I do | If your answer is no, go to Q.N. 319 |
| 318 | How many times the problem occurs in the past one month? | 1.Occasionally ( one or two times ) ___  2. Sometimes ( three to four times) ___  3. Many times ( above ten times) ___ |  |
| 319 | Yesterday, was there unusual meal in your house or neighbor because it was a special day | 1. Yes 2. No |  |

**Section two**

**Questions referring children less than 24 months of age**

4. General information and child feeding practices

| **S.N** | **Question** | **Response** |  |
| --- | --- | --- | --- |
|  | Sex of the child | Male _____  Female _____ |  |
|  | The relationship of the respondent with the child | - - - 1. Biological mother ____       2. Biological father ____       3. Step mother ____       4. Step father _____       5. Other (specify) ______________ |  |
|  | Age of the child | _____ Months |  |
|  | Date of birth | ___/___/_____ (DD/MM/YYYY) |  |
|  | Date of birth is confirmed with vaccination card or birth certificate | Yes ____  No ____ |  |
|  | Place of birth | 1. Home _____ 2. Health institution ( specify) ______________________ 3. Other (specify) _____________ |  |
|  | Is your child currently breast-feeding? | 1. Yes _____ 2. No _____ |  |
|  | If no, what is the reason | - - - 1. Mother’s health related _____       2. Mother’s job related_____       3. Child health related_____       4. Satisfied with breast milk (age) ____       5. Separated from mother ____       6. Current pregnancy ____       7. Other (specify) ___________________ |  |
|  | When did you start breast feeding to the child | 1. Immediately after birth _____ 2. Within one hour after birth ____ 3. After one hour from birth _____ 4. After 24-hour from birth _____ 5. Other(specify) ______________ |  |
| 410 | Did you give any Pre-lacteal feed to the child? | 1. Yes _____ 2. No _____ |  |
| 411 | If yes, what was given? | 1. Formula milk _________ 2. Cow’s milk ____________ 3. Sugar and water_________ 4. Other(specify) _________________ |  |
| 412 | Did you use bottle feeding to your child? | 1. Yes _____ 2. No _____ |  |
| 413 | Is this child stated complementary feeding | 1. Yes ______ 2. No ______ |  |
| 414 | If yes, at what age the child started complementary feeding? | At ______________months |  |

- - - 1. Meal frequency questions for the child

|  | For the following animal-source foods, indicate with a checkmark (√) in the category that best describes the frequency with which your child ate that particular food item during the past three months. | | | | | | |
| --- | --- | --- | --- | --- | --- | --- | --- |
|  | **Food item** | **Always**  **(**least once per day**)** | **Often**  **(**one to six times per week**)** | **Sometimes**  **(**one to three times per month**)** | **Rarely (**less than once per month**)** | **Never**  **(**never ate in the past three months**)** | **Comment if any** |
| 501 | Milk and milk products |  |  |  |  |  |  |
| 502 | Eggs |  |  |  |  |  |  |
| 503 | Organ meat |  |  |  |  |  |  |
| 504 | Flesh meat |  |  |  |  |  |  |
| 505 | Fish |  |  |  |  |  |  |
| Other animal-source foods not listed | |  |  |  |  |  |  |
| 1 |  |  |  |  |  |  |  |
| 2 |  |  |  |  |  |  |  |
| 3 |  |  |  |  |  |  |  |

**Section Three**

**Questions about the mother**

- - - 1. Reproductive characteristics and general information of the index mother

| 601 | Age of the mother | _______ years |  |
| --- | --- | --- | --- |
| 602 | What is highest completed level of your education? | 1. No schooling at all _____ 2. Completed Grade _______ 3. Certificate/ diploma level _____ 4. Degree and above____ |  |
| 603 | Are you employed | Yes ______ No _______ |  |
| 604 | Total number of pregnancy | ___________ |  |
| 605 | Age at first pregnancy | ___________ years old |  |
| 606 | Currently pregnant? | 1. Yes ______ 2. No ______ 3. I don’t know ______ |  |

- - - 1. Meal frequency questions for the mother

|  | For the following animal-source foods, indicate with a checkmark (√) in the category that best describes the frequency with which you ate that particular food item during the past three months. | | | | | | |
| --- | --- | --- | --- | --- | --- | --- | --- |
|  | **Food item** | **Always**  **(**at least once per day**)** | **Often**  **(**one to six times per week**)** | **Sometimes**  **(**one to three times per month**)** | **Rarely (**less than once per month**)** | **Never**  **(**never ate in the past three months**)** | **Comment if any** |
| 701 | Milk and milk products |  |  |  |  |  |  |
| 702 | Eggs |  |  |  |  |  |  |
| 703 | Organ meat |  |  |  |  |  |  |
| 704 | Flesh meat |  |  |  |  |  |  |
| 705 | Fish |  |  |  |  |  |  |
| Other animal-source foods not listed | |  |  |  |  |  |  |
| 1 |  |  |  |  |  |  |  |
| 2 |  |  |  |  |  |  |  |
| 3 |  |  |  |  |  |  |  |

**Thank you!**
